# Cigarette Smoke Exposure Leads to Organic and Mineral Bone Component Changes: The Importance of Rho Kinase Function in These Events

**DOI:** 10.3390/cells14070503

**Published:** 2025-03-28

**Authors:** Alex Ferreira da Silva, Franciele Jesus Lima, Alyne Riani Moreira, Cintia do Nascimento Silva, Ivone Braga de Oliveira, Alexandra Fernandes Callera, Ana Luiza Porfirio, Luan Henrique Vasconcelos Alves, Iolanda de Fátima Lopes Calvo Tibério, Ana Paula Pereira Velosa, Vanda Jorgetti, Walcy Rosolia Teodoro, Fernanda Degobbi Tibério Quirino Dos Santos Lopes

**Affiliations:** 1 Laboratory of Experimental Therapeutics (LIM-20), Department of Medicine, School of Medicine, University of São Paulo, São Paulo 01246-903, Brazil; alex-ferreira@usp.br (A.F.d.S.); fisiofransilva@gmail.com (F.J.L.); riane84@yahoo.com.br (A.R.M.); cintianascimento777@gmail.com (C.d.N.S.); alexandra.callera@gmail.com (A.F.C.); ana.luiza.ps.alp@gmail.com (A.L.P.); luan.vasc05@hotmail.com (L.H.V.A.); iocalvo@uol.com.br (I.d.F.L.C.T.); 2 Laboratory of Renal Physiopathology (LIM-16), Department of Medicine, School of Medicine, University of São Paulo, São Paulo 01246-903, Brazil; ivoneol@usp.br (I.B.d.O.); vandajor@usp.br (V.J.); 3 Rheumatology Division (LIM-17), School of Medicine, University of São Paulo, São Paulo 01246-903, Brazil; ana.velosa@hc.fm.usp.br (A.P.P.V.); walcy.teodoro@fm.usp.br (W.R.T.); 4 Thoracic Surgery Research Laboratory (LIM-61), Division of Thoracic Surgery, Instituto do Coracao do Hospital das Clínicas da Faculdade de Medicina da Universidade de São Paulo, São Paulo 05403-000, Brazil

**Keywords:** Rho kinase inhibitor, smoking, mineral components, osteoblast activity

## Abstract

Aberrant Rho-associated kinase function could be associated with increased bone fragility. Since cigarette smoke (CS) exposure promotes the increase in bone fragility due to changes in bone tissue components, this study aimed to investigate how CS exposure could modulate the Rho kinase-associated bone structural changes. Mice were assigned to four groups: control; smoke; control with Rho kinase inhibitor administration; and smoke with a Rho kinase inhibitor. Bone samples were obtained to assess bone histomorphometry analysis, type I collagen composition, and MEPE expression in trabeculae. We observed that CS exposure induced decreased trabecular and osteoid thickness. A concomitant increase in the osteoclastic and erosion surfaces and a decrease in the mineralization surface were observed. Additionally, CS exposure decreased the type I collagen and MEPE expression. Rho kinase inhibitor administration recovered the bone mineralization and the collagen type I deposition. Conclusions: CS exposure increases Rho kinase activity in bone cells, leading to structural changes. The administration of a Rho GTPases inhibitor partially reverses these effects, likely due to the recovery in osteoblast activity.

## 1. Introduction

Smoking remains the leading global cause of preventable deaths, resulting in more than 8 million deaths each year, mostly attributed to respiratory diseases, heart attacks, and vascular diseases [1]. Additionally, extensive evidence has shown that smoking worsens the bone remodeling process, inducing bone loss and leading to reductions in bone length, weight, and mineral density, as well as an increase in osteoclastogenesis and the inhibition of osteoblastogenesis [2,3,4].

Previously, we showed important changes in bone tissue components in an experimental model of cigarette smoke (CS) exposure, such as the worsening of bone mineralization and a decrease in collagen type I deposition, leading to bone fragility. Also, we showed the effects of smoking on the increase in osteoblast apoptosis with a concomitant reduction in type I collagen deposition, even in former smokers who had quit smoking for at least ten years [3].

Osteoblasts are bone cells responsible for producing the extracellular matrix (ECM) bone components. These cells produce an extracellular matrix (ECM) principally composed of fibrillar collagen, in which they subsequently deposit hydroxyapatite (HA). This process occurs efficiently if the cells play an efficient migration and adhesion through the bone tissue [5].

Rho GTPases are intracellular signaling molecules recognized by orchestrating the signaling pathways that regulate cell migration, adhesion, and cytokinesis [6]. Rho-associated kinase (Rho kinase, ROCK, or Rok-alpha) is an effector of small GTPases, mediating cell motility and contractility [7]. Rho kinase (ROCK, Rho-associated protein kinase) regulates the cytoskeleton in various cell types. It is an enzyme activated by signals from the RhoA protein, which plays a key role in intracellular signaling processes that regulate the actin cytoskeleton. In bone cells, such as osteoblasts, Rho kinase modulates the organization of actin filaments, promoting their contraction and reorganization. It influences the formation of stereocilia (cell adhesion structures) and cell morphology, regulating focal adhesion. These adhesions are crucial for attachment to the bone matrix and for transmitting signals to other cells. Thus, Rho kinase activation enhances the stabilization of these adhesions, impacting osteoblast function and bone formation [8,9,10].

Aberrant Rho-associated kinase function appears to play a major role in different diseases such as cancer, vascular diseases, and asthma [11,12]. The signaling pathways regulated by these molecules need to be further elucidated [12]. Shi et al. demonstrated that Rhoa/ROCK activation may be a mechanism underlying aging-associated bone loss [13]. Moreover, Prowse et al. showed that the inhibition of RAC and ROCK signaling ameliorates osteoblast adhesion by modulating cytoskeletal tension, differentiation, and mineralization on titanium topographies [14]. Considering that smoking induces alterations in the bone ECM composition and impairs bone mineralization [3] and that aberrant Rho kinase activity has been shown to play a crucial role in modulating bone cell metabolism, this study aims to evaluate the effects of Rho kinase on the deposition of organic and mineral bone components in an experimental model of cigarette smoke exposure.

## 2. Materials and Methods

### 2.1. Experimental Groups

This study was approved by the Ethics Committee on Animal Studies of the School of Medicine, University of São Paulo (Animal Use Ethics Committee—CEUA—Project Number 1740/2021). Male C57BL/6 mice (6 to 8 weeks old), with an average weight of 26 g, were provided by the Central Animal Facility of the School of Medicine of the University of São Paulo. All animals received care in accordance with the Guide for the Care and Use of Laboratory Animals.

Animals were divided into four experimental groups as follows:

- Control Group (C). Animals remained in the animal facility receiving filtered air ventilation (*n* = 9).
- Smoke Group (S). Animals were exposed to cigarette smoke for 6 weeks (*n* = 9).
- Control and Rho Kinase Inhibitor Group (C-RI). Animals remained in the animal facility receiving filtered air ventilation and were treated with intraperitoneal injections of a Rho kinase inhibitor (*n* = 8).
- Smoke and Rho Kinase Inhibitor Group (S-RI). Animals were exposed to cigarette smoke for 6 weeks and treated with intraperitoneal injections of a Rho kinase inhibitor (*n* = 8).

### 2.2. Cigarette Smoke Exposure Model

**Figure 1:**
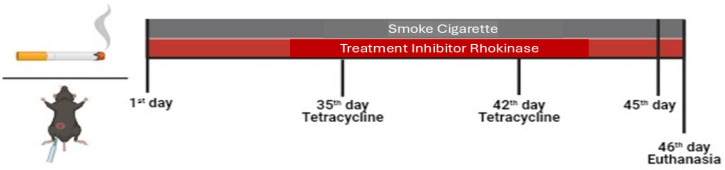
Protocol timeline illustration.

Animals in the smoke group and the smoke RI group were exposed to cigarettes twice a day, five times a week, for six weeks (Figure 1), as previously described. Animals were exposed to 12 ± 1 commercially filtered cigarettes (0.8 mg of nicotine, 10 mg of tar, and 10 mg of CO per cigarette), resulting in a total particulate matter concentration of 354.8 ± 50.3 μg/m^3^/day. Carbon monoxide (CO) levels ranged from 250 to 350 parts per million (ppm) by adjusting the flow rate into the exposure box [2,4].

### 2.3. Treatment with a Rho Kinase Inhibitor and the Application of Tetracycline

The animals were administered treatment with the Rho kinase inhibitor once daily, one hour prior to exposure, five days per week, for a duration of six weeks. The injection was delivered via intraperitoneal administration using the inhibitor Y-27632 (Cayman, Biological Company, Ann Arbor, Michigan, USA) at a dose of 10 mg/kg [15,16].

Tetracycline, an antibiotic from the macrolide group, deposits on the bone surface, allowing for the evaluation of dynamic parameters of bone formation and mineralization. All groups received one dose of Tetracycline (Terramicina LA 20 mL, Zoetis, São Paulo, Brazil) on the 35th and 42nd days of the protocol to ensure that the marking effect remained until the day of euthanasia. The administration was performed via intramuscular injection (0.02 mg/kg) using an insulin syringe.

### 2.4. Euthanasia for Tissue Collection

Upon completion of the exposure and treatment protocol for the animals, we performed the euthanasia by intraperitoneal injection of sodium thiopental at 70 mg/mL. After anesthesia induction, a laparotomy was performed to access the inferior vena cava for exsanguination. Subsequently, tissue removal was carried out.

The bilateral tibiae of each animal were extracted to measure histomorphometric parameters. These assessments were conducted in the metaphyseal bone region, below the lowest point of the growth plate (below the primary spongiosa) and internal to the lateral cortex, excluding the cortical bone. This selected area consists of trabecular-rich cancellous bone (secondary spongiosa), which contains structural components and the organic portion of the matrix (collagen I). The right tibia was used for histomorphometry and MEPE quantification, while the left tibia was processed to prepare histological slides. Additionally, both the right and left femurs were extracted to prepare tissue macerates. These bones provide more structure and tissue compared to others, allowing for the collection of a greater amount of bone matrix components.

### 2.5. Bone Histological Preparation

The left tibia of each animal underwent surgical extraction and cleaning of adjacent tissues. Subsequently, the collected tissues were fixed in 10% buffered formalin for 24 h, decalcified with 7% nitric acid solution for 3 days, washed in running water for 20 min, immersed in distilled water, and finally placed in buffered 10% formalin. After this period, the bone samples were immersed in 70% alcohol for two days and embedded in paraffin. Sections with a thickness of 3 μm were prepared, with 50 μm spacing between them, for use in histological staining techniques. Four slides were prepared for each animal, which is necessary for subsequent standardization of specific immunohistochemical markers.

### 2.6. Immunofluorescence for Type I Collagen

We evaluated the proportions of type I collagen present in the tibia metaphysis of the mice using the immunofluorescence technique. Images were obtained using a photographic camera (Olympus Co., St. Laurent, QC, Canada) attached to the Olympus BX-51 microscope (Olympus BX51, Olympus Co., Tokyo, Japan). These images were digitized (Oculus TCX, Coreco, Inc., St. Laurent, QC, Canada). Subsequently, these images are processed using the Image-Pro Plus 6.0 software (Media Cybernetics, Rockville, MD, USA), which stains the immune labeled fibers in green, showing the percentage of labeled collagen relative to the total trabecular area.

The tibia sections were prepared on polarized slides, which were silane coated and had positive/negative charges to enhance tissue adherence after deparaffinization (in heated xylene). Rehydration in ethanol washes was followed by water. After the washes, the sections underwent digestion with porcine gastric pepsin diluted in acetic acid. The combination of gastric pepsin and acid ensures better digestion for bone tissue. After incubation, the sections were subjected to a cycle of washes with running water and a cycle of distilled water and were subsequently washed with PBS three times for 10 min each. After this process, the antigenic sites were expected to be exposed.

For the immune detection of type I collagen, nonspecific binding sites were blocked with 5% bovine serum albumin (BSA) in PBS for 30 min at room temperature (Figure S1). Subsequently, the samples were incubated overnight at 4 °C with rabbit polyclonal anti-type I collagen antibodies (1:100, Rockland Immunochemicals, Gilbertsville, PA, USA) diluted in PBS solution. The samples were washed in PBS containing 0.05% Tween 20 and incubated for 60 min at room temperature with Alexa 488-conjugated goat anti-rabbit IgG antibodies (Invitrogen, Thermo Fisher Scientific, Waltham, MA, USA) diluted in Evans Blue (1:200).

### 2.7. Histomorphometry

For histomorphometric analyses, the right tibiae of each animal were collected, surgically extracted, cleaned of adjacent tissues, and subsequently immersed in 70% alcohol. They were prepared in a methyl methacrylate embedding medium for undecalcified bone, following a previously described technique.

Histomorphometric analyses were performed on histological sections of 5 μm thickness, which were stained with 0.1% toluidine blue, pH 6.4, and coverslipped with Entellan H mounting medium (Merck, Darmstadt, Germany). A PolycutS equipped with a tungsten blade (Leica, Heidelberg, Germany) was used.

Static, structural, and dynamic parameters were measured in 9 to 10 fields within the proximal tibia metaphysis using a standardized image analysis method. The structures of interest were manually marked using a microscope (Nikon, Labophot-2A, Tokyo, Japan), cursor, and digitizing tablet. The final calculation of the parameters was performed using specific histomorphometry software—OsteoMeasure (OsteoMetrics, Inc., Atlanta, Georgia, USA). All parameters were measured according to the guidelines of the *American Society of Bone Mineral Research Histomorphometry Nomenclature Committee*.

Histomorphometric parameters are divided into structural and remodeling parameters, with the latter further subdivided into resorptive and formative parameters. Structural parameters include trabecular bone volume to total bone volume ratio (BV/TV) and trabecular thickness (Tb.Th) (Table 1).

Remodeling parameters include resorptive parameters, such as eroded surface area (ES/BS) and osteoclastic surface (Oc.S/BS), and formative parameters such as osteoid thickness (O.Th), osteoid surface area (OS/BS), osteoblastic surface (Ob.S/BS), mineralizing surface (MS/BS), mineral apposition rate (MAR), mineralization lag time (MLT), and bone formation rate (BFR/BS) (Table 1).

All analyses were performed with the slides blinded to the evaluator. The indices were all reported using the nomenclature of the American Society for Bone and Mineral Research.

### 2.8. Immunohistochemistry for MEPE

For the quantification of positive cells for MEPE, the same tissue described previously in the histomorphometry analysis was used, as calcified bone tissue was required to preserve the maximum number of cells present in the matrix, primarily osteocytes, which produce this protein.

To perform the immunohistochemistry technique, the methacrylate was removed from the tissue using a 1:1 solution of xylene and chloroform for 30 min under agitation. After gel removal, the slides were immersed in a xylene bath (1×) for 1 min, followed by ethanol baths at different concentrations (100%, 96%, 70%, and 50%) for 20 s each, and they were finally washed once with distilled water for 5 min. Partial decalcification of the tissue was performed using 1% acetic acid for 15 min under agitation, followed by a 5 min wash with distilled water to remove excess acid. To clean the background and block nonspecific binding sites, endogenous peroxidase was blocked for 15 min in the dark (20 mL of 3% hydrogen peroxide + 180 mL of methanol). Additionally, avidin and biotin were blocked (Vector Avidin/Biotin BLOCKING KIT), and a biotinylated horse anti-goat serum (diluted 1:50 in 0.5% BSA) was used (Figure S1). To prevent tissue drying, the samples were isolated using a hydrophobic pen and incubated overnight in a humid chamber at 4 °C with the primary antibody (anti-hMEPE 1:100 in PBS, R&D System, AF3140) in a humid chamber in the refrigerator.

After the incubation, the excess primary antibody was removed, and the tissue was washed three times with PBS containing 0.05% Tween 20 for 5 min each. Next, the tissue was incubated with a secondary antibody (anti-goat diluted in 0.5% BSA—Vector/Lot. ZH0716) for 45 min at room temperature, followed by washing and incubation with the ABC complex (1:100—HRP, Vector) for 30 min at room temperature. The reaction was developed using DAB and counterstained with Mayer’s hematoxylin (with specific development times for each reaction), and coverslips were mounted with glycerol gel. We assessed the proportions of MEPE present in the tibial metaphysis of the mice by quantifying the number of positive cells (actively producing MEPE) and total cells (previously produced MEPE) using images obtained from a photographic camera (Olympus Co., St. Laurent, QC, Canada) attached to the Olympus BX-51 microscope (Olympus BX51, Olympus Co., Tokyo, Japan). These images were then digitized (Oculus TCX, Coreco, Inc., St. Laurent, QC, Canada). Subsequently, these images were processed using the software Image-Pro Plus 6.0 (Media Cybernetics, Rockville, Maryland, USA), which allowed the MEPE ^+^ osteocyte density to be calculated using the total number of positive cells relative to the total cell count, corrected for the total area of the trabecula.

The statistical analysis was performed using SigmaStat 11.0 software (SPSS Inc., Chicago, IL, USA). Histomorphometric measurements and protein quantification data were analyzed using two-way ANOVA, as we evaluated two factors (smoke and inhibitor administration), followed by Holm–Sidak post hoc analysis due to the parametric distribution of the data. The results were presented as mean ± SE, and a *p*-value < 0.05 was considered statistically significant.

### 2.9. Ethical Declarations

The manuscript does not contain clinical studies or patient data. All applicable institutional and/or national guidelines for the care and use of animals were followed.

The Research Ethics Committee of the Faculty of Medicine of the University of São Paulo, in a session on 8 December 2021, approved Research Protocol n° 1740/2021 entitled a “Study of the effects of treatment with a specific Rho kinase inhibitor on bone metabolism in a cigarette smoke exposure model”, which involved the use of 55 mice provided by the Faculty of Medicine of the University of São Paulo.

It is the responsibility of the researcher to prepare and submit the final research report to the CEP-FMUSP, in accordance with legal procedures for the scientific use of animals (Law No. 11,794, 8 October 2008).

## 3. Results

### 3.1. Structural Parameters

**Figure 2:**
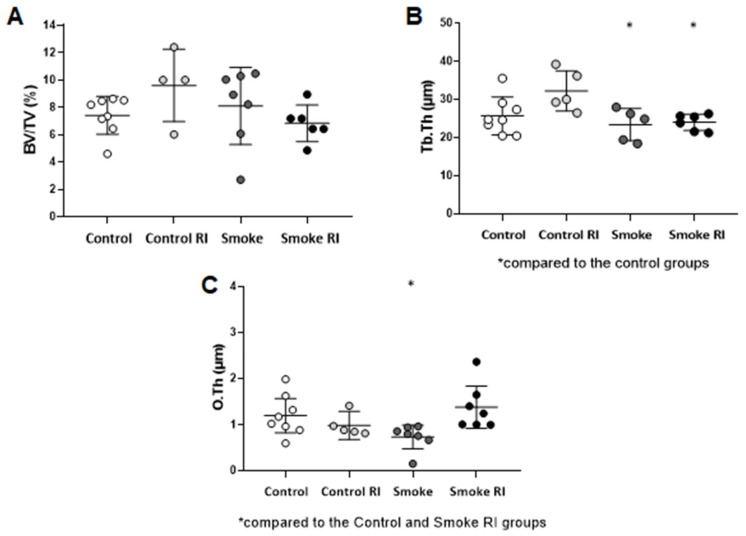
Mean of BV/TV (ratio of trabecular bone volume to total bone volume) (**A**), Tb.Th (trabecular thickness) (**B**), and O.Th (osteoid thickness) (**C**). In graph (**B**), there is a statistically significant decrease in the smoke and smoke RI groups (*p* < 0.009) compared to the control groups. In graph (**C**), there is a statistically significant decrease in the smoke (*p* < 0.010) compared to the control and smoke RI groups. Values are expressed as mean ± standard error.

In structural parameters, the trabecular thickness and organic components showed a loss in the evaluation parameters. There was a reduction in Tb.Th in the smoke and smoke RI groups compared with the control groups (Figure 2B, *p* < 0.009). The analysis of the O.Th parameter demonstrated a reduction in the smoke group compared with both the control and smoke RI groups (Figure 2C, *p* < 0.010). The analysis of BV/TV was similar between the groups (Figure 2A).

### 3.2. Remodeling Parameters

#### Resorptive Indices

**Figure 3:**
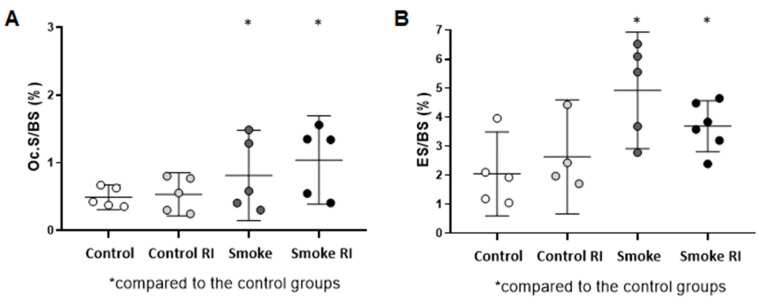
Mean of Oc.S/BS (osteoclastic surface) (**A**) and ES/BS (erosion surface) (**B**). In graph (**A**), there was an increase in the osteoclastic surface in both the smoke groups (*p* < 0.036) compared to the controls. In graph (**B**), there was an increase in erosion surface in both the smoke groups (*p* < 0.003) compared to the controls, and it was notably higher in the smoke group. Values are expressed as mean ± standard error.

In the analysis of resorptive indices, we observed an important increase in osteoclastic surface (Oc.S/BS) and eroded surface (ES/BS) in both the smoke RI and smoke groups compared to the control groups (Figure 3A, *p* < 0.036; Figure 3B, *p* < 0.003).

### 3.3. Formative Indices

**Figure 4:**
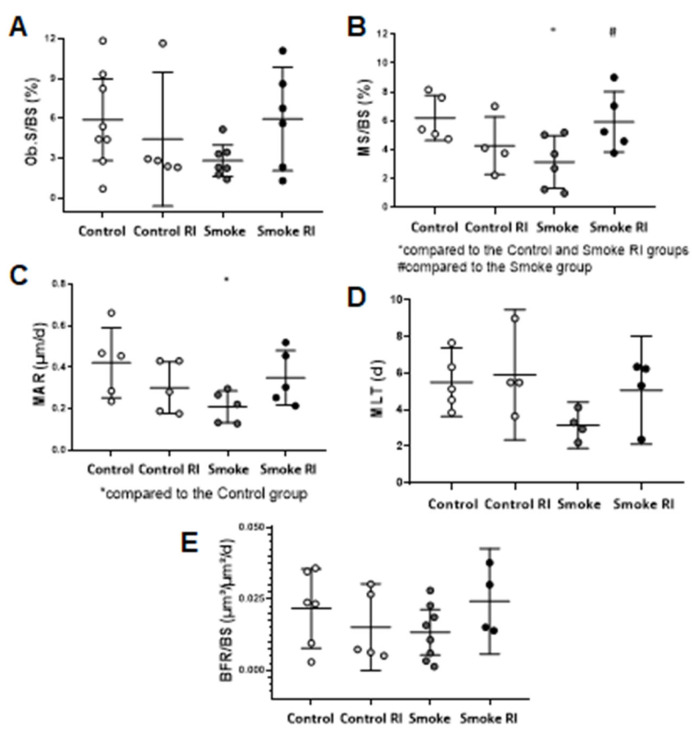
Mean values of Ob.S/BS (osteoblastic surface) (**A**), MS/BS (mineralizing surface) (**B**), MAR (mineral apposition rate (**C**), MLT (mineralization lag time) (**D**), and BFR/BS (bone formation rate) (**E**). In graph (**A**), there were no differences in the osteoblastic surface when comparing the experimental groups. In graph (**B**), there was a statistically significant decrease in the mineralizing surface in the smoke group (*p* < 0.016) compared to the control and smoke RI groups. Also, there was an increase in the mineralizing surface in the smoke RI group (*p* < 0.026) compared to the smoke group. In graph (**C**), there was a statistically significant decrease in the mineral apposition rate in the smoke group (*p* < 0.020) compared to the control group. In graph (**D**), there were no differences in the mineralizing lag when comparing the experimental groups. In graph (**E**), there were no differences when comparing bone formation between the experimental groups. Values are expressed as mean ± standard error.

The results of bone mineralization (surface mineralization of cancellous bone—MS/BS) showed a decrease in the smoke group compared to the control group, while an increase was observed in the smoke RI group compared to the smoke group (Figure 4B, *p* < 0.016, *p* < 0.026). Additionally, we observed a decrease in the mineral apposition rate (MAR) in the smoke group compared to the control group (Figure 4C, *p* < 0.020). We also evaluated the osteoblastic surface (Ob.S/BS), mineralization lag time (MLT), and bone formation rate (BFR/BS). However, no statistically significant differences were found (Figure 4).

### 3.4. Proportion of Type I Collagen and MEPE

**Figure 5:**
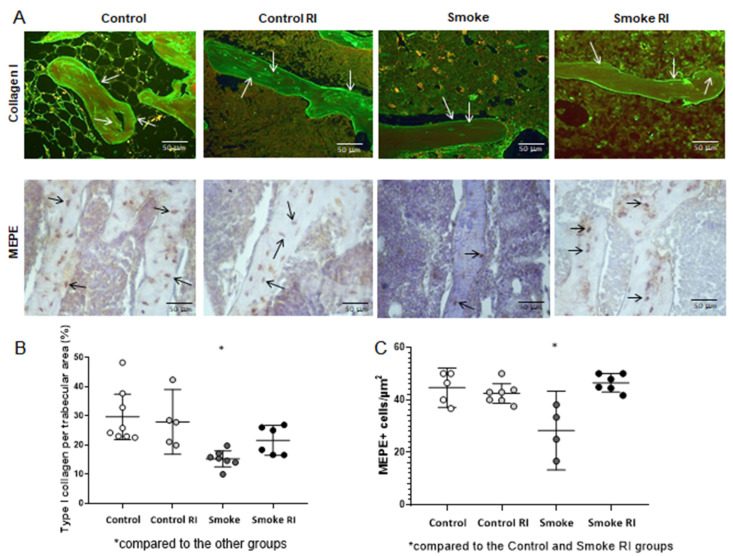
Immunofluorescence for type I collagen and immunohistochemistry for MEPE (panel (**A**)) of the control, control RI, smoke, and smoke RI groups in bone tissue. In panel (**A**), an illustrative image of the expression of collagen type I and MEPE^+^ cells of each group is shown. In graph (**B**), there was a significant reduction in the expression of type I collagen (arrows) in the smoke group (*p* < 0.001) compared to the other groups. In graph (**C**), when analyzing MEPE, there was a significant reduction in the amount of MEPE^+^ cells per trabecular area in the smoke group (*p* < 0.001) compared to the control and smoke RI groups. Values are expressed as mean ± standard error.

These analyses showed a reduction in type I collagen compared to the control groups and smoke RI group (*p* < 0.001). The smoke group has demonstrated a decrease in MEPE compared to both the control and smoke RI groups (*p* < 0.001) (Figure 5). Organic proteins showed a decrease in type I collagen expression in the smoke group compared to the other groups (Figure 5B, *p* < 0.001). Additionally, we observed a reduction in MEPE^+^ cells per trabecular area in the smoke group compared to the control and smoke RI groups (Figure 5C, *p* < 0.001).

## 4. Discussion

In this study, we showed that bone tissue component changes induced by CS exposure could be partially attributed to a Rho-associated kinase aberrant activity.

Regarding structural parameters, we observed that CS exposure led to a decrease in trabecular and osteoid thickness (Tb.Th and O.Th, respectively), and Rho kinase inhibitor administration reversed the decrease in O.Th. These findings align with the analysis of type I collagen in the trabecular area. There was an important recovery of collagen type I deposition in animals exposed to CS that received Rho kinase administration compared to animals exposed to CS only.

The analysis of bone resorptive indices (Oc.S/BS; ES/BS) reinforced the impact of smoking on increased bone resorption. Although Rho kinase inhibitor administration did not attenuate the effects on resorptive indices, its administration showed important effects on formative parameters. A decrease in MS/BS and MAR indices was observed only in the smoke group, whereas the smoke RI group recovered these indices values, reaching amounts similar to those observed in the control groups.

The matrix extracellular phosphoglycoprotein (MEPE) plays a crucial role in the bone mineralization process [17]. Previous studies have shown that in MEPE null mice, the ablation of MEPE leads to increased bone mass due to a higher number and activity of osteoblasts [17,18,19,20]. In this study, MEPE^+^ cell evaluation reinforces previous findings since the bone trabecular area results suggest that the Rho kinase inhibitor recovers bone mineralization in animals exposed to CS. Given that we also demonstrated the effects of this inhibitor in the collagen type I deposition recovery, Rho kinase inhibitor administration may enhance osteoblast activity in animals exposed to CS. Furthermore, these results align with improvements observed in bone formation parameters (MS/BS and MAR indices).

The effects of smoking on increased bone fragility, higher fracture incidence [21], osteoporosis, especially in women [22], and delayed bone healing [23] have been extensively described. Clinical studies have demonstrated that smokers experience a reduction in bone volume and mineral density (BMD). However, despite strong clinical evidence of smoking’s impact on bone health, the specific mechanisms induced by smoking and how they impair bone turnover remain unclear [22,23,24,25,26]. Our results reinforce the previous findings of our group [2,3,4], as we demonstrated that smoke leads to an increase in resorptive parameters, concomitant with the decrease in part of the formative and structural parameters. Furthermore, we advanced our understanding of the effects of CS on bone cells by investigating intracellular signaling molecules that regulate the actin cytoskeleton of bone cells. Rho GTPases and their downstream effectors, such as Rho kinases, regulate cell proliferation, adhesion, migration, and apoptosis by modulating cell shape, cytoskeletal dynamics, and cell–cell contacts [27,28]. The inhibition of Rho kinase has been shown to enhance the survival of stem cells from various sources [29] and may also suppress themtheir [30]. Also, in vivo and in vitro studies attested to the effects of Rho kinase on the actin–myosin cytoskeleton, contributing to increased apoptosis in different cell types [31]. During the repair and regeneration process, cell migration to the injury site and survival are essential for effective wound healing [30]. In bone tissue, osteoblasts play a crucial role in the deposition of organic and inorganic components.

Since our findings demonstrate that Rho-associated kinase inhibition enhances collagen type I deposition and increases parameters that describe the occurrence of bone mineralization, even in animals exposed to CS, we propose that CS exposure induces aberrant Rho-associated kinase activity, which may compromise osteoblast migration and adhesion as well as promote apoptosis. The effects of smoking on inducing aberrant Rho kinase activity have been most described in respiratory diseases, particularly in the smooth muscle surrounding the airways [32,33,34,35,36,37]. To our knowledge, this is the first study that highlights the effects of smoking on Rho kinase activity in bone cells.

## 5. Conclusions

In this study, we demonstrated that CS exposure increases Rho kinase activity in osteoblasts and osteoclasts, leading to structural changes in both organic and inorganic bone components. The administration of a Rho GTPase inhibitor partially reversed these events, recovering the collagen type I deposition and enhancing the bone mineralization process, likely by promoting osteoblast activity.

## Figures and Tables

**Table 1:** Description of histomorphometric parameters.

Histomorphometric parameters		
Structural parameters:	Abbreviation	Unit
Ratio of trabecular bone volume to total bone volume	BV/TV	%
Trabecular thickness	Tb.Th	μm
Resorptive remodeling parameters:		
Area of eroded surface	ES/BS	%
Osteoclastic surface	Oc.S/BS	%
Formative remodeling parameters:		
Osteoid thickness	O.Th	μm
Osteoblastic surface	Ob.S/BS	%
Mineralizing surface	MS/BS	%
Mineral apposition rate	MAR	μm/d
Mineralization lag time	Mlt	d
Bone formation rate (S)	BFR/BS	μm^3^/μm^2^/d

## Data Availability

All data are available for evaluation if requested.

## Supplementary Materials

The following are available online at https://www.mdpi.com/article/10.3390/cells14070503/s1, Figure S1: illustration of negative and positive controls for type I collagen and MEPE^+^ cells
