# Supplementary material for: Cigarette Smoke Exposure Leads to Organic and Mineral Bone Component Changes: The Importance of Rho Kinase Function in These Events
Source: Cells. 2025 Mar 28;14(7):503. doi: 10.3390/cells14070503 (PMC11987806; doi:10.3390/cells14070503)
Supplement: Supplementary file 1 [file cells-14-00503-s001.zip › cells-3481791-supplementary.pdf]

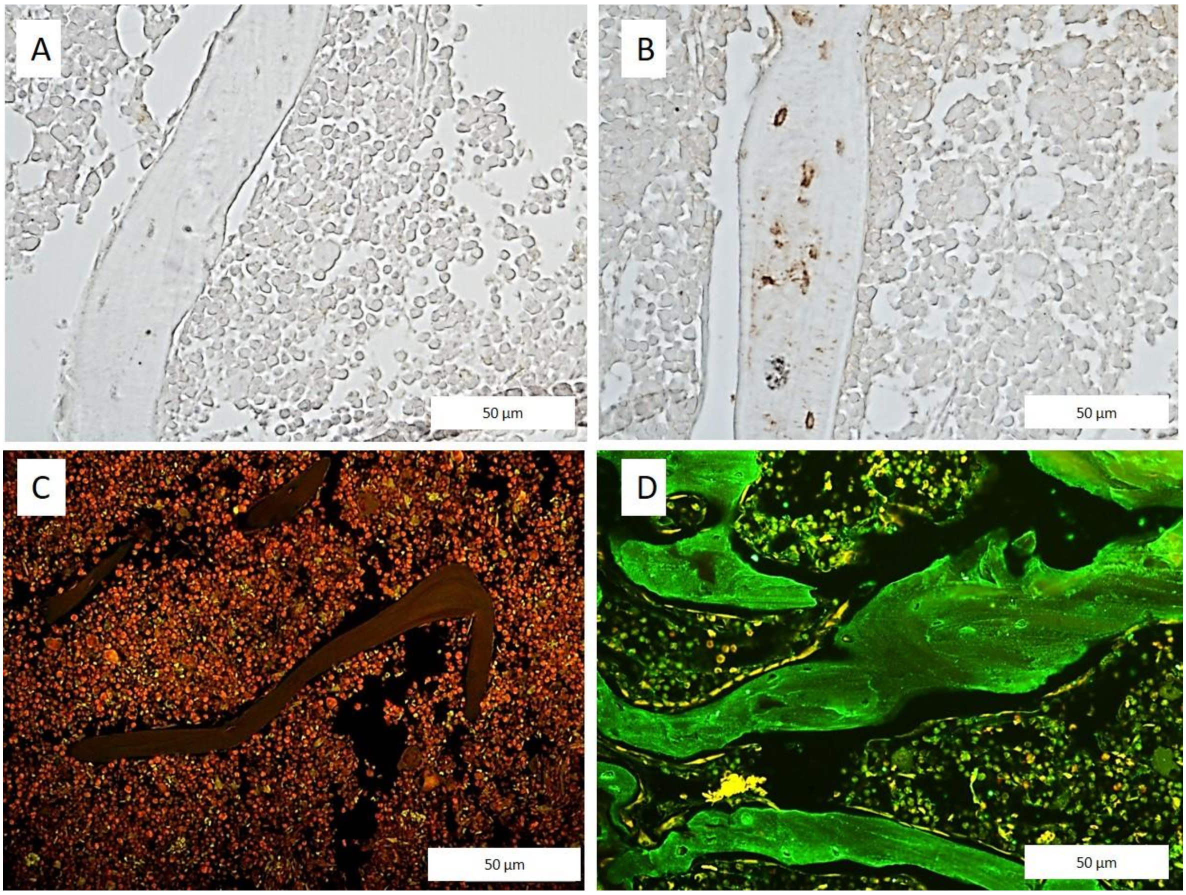

Figure S1: illustration of negative and positive controls for type Ⅰ collagen and MEPE+ cells. Panel A shows an illustrative image of the negative control for MEPE+ cells. In Panel B is the exemplary image of the positive control for MEPE+ cells. In Panel C is an image of the negative control for type Ⅰ collagen. In Panel D is an image of the positive cell for type Ⅰ collagen.
